# Bartlett on Fevers

**Published:** 1848-05

**Authors:** 


*Bartlett on Fevers.*—We owe an apology, not only to the author and publishers of this work, but also to our readers, for delaying critical notice of it. We have intended to prepare an extended review, but the pressure of various duties has prevented the execution of this design. We shall devote some space to it in our next number.

